# Holography-guided procedural planning for modifying Venus *P*-valve implantation technique in patients with left pulmonary artery stents: a case-series

**DOI:** 10.3389/fcvm.2024.1378924

**Published:** 2024-05-13

**Authors:** Angelo Fabio d’Aiello, Laura Schianchi, Francesca Bevilacqua, Paolo Ferrero, Angelo Micheletti, Diana Gabriela Negura, Giulia Pasqualin, Massimo Chessa

**Affiliations:** ^1^ Adult Congenital Heart Disease (ACHD) Unit, IRCCS Policlinico San Donato, San Donato Milanese, Milan, Italy; ^2^ Vita-Salute San Raffaele University, Milan, Italy

**Keywords:** mixed reality, holographic models, procedural planning, pulmonary valve, Venus *P*-valve, self-expandable valves, left pulmonary artery stents

## Abstract

**Background:**

Venus *P*-valve™ (Venus Medtech, Hangzhou, China) is a self-expandable bioprosthetic valve that can be transcatheter-implanted in native right ventricular outflow tract (RVOT) patients. Valve implantation is technically challenging. Due to the implantation technique, left pulmonary artery (LPA) stents represent a relative contraindication to Venus *P*-valve. In this case series, we describe our experience in implanting Venus *P*-valve in patients with previous LPA stents and the use of holographic models to facilitate procedural planning.

**Methods and results:**

From January to October 2023, 17 patients were scheduled for Venus *P*-Valve implantation. 16/17 (94%) patients were successfully implanted. 3/16 (18.7%) patients underwent Venus *P*-valve implantation with LPA stents. All patients underwent pre-operative CT scan. CT data set were employed to create three-dimensional (3D) holographic models (Artiness, Milan, Italy) of the entire heart, which were useful to plan valve implantation with a modified technique. Procedural success rate was 100%. No procedural complications occurred. All three patients presented good haemodynamic and angiographic results at discharge and follow-up visits.

**Conclusion:**

This case-series underscores the feasibility of Venus *P*-valve implantation in patients with previous LPA stents. The use of holographic models facilitated procedural planning in these challenging anatomical scenarios.

## Background

**Figure 1:**
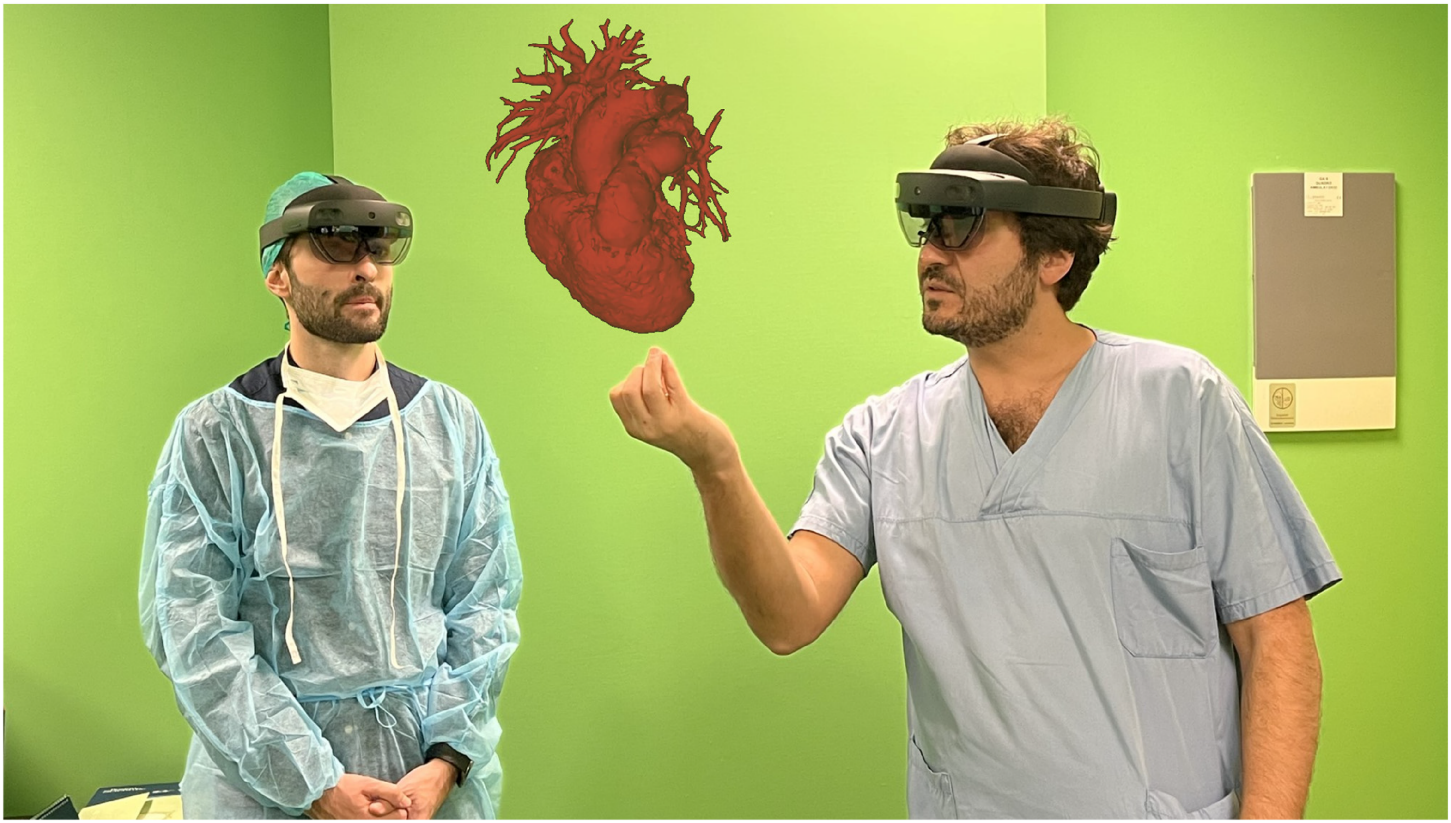
Holography-guided pre-procedural planning.

Pulmonary valve regurgitation (PR) is common in patients with congenital heart disease (CHD) (1). Severe PR and RVOT dilatation can compromise right ventricular hemodynamics by the imposition of a volume and pressure load (2, 3). With improved CDH survival (1), the complications related to right ventricular overload that result from PR have come to light, including arrythmias and right sided heart failure (3). Historically, these issues were managed through multiple surgical interventions, with increased risk of related morbidities and increased mortality (2, 4–6). Nowadays, transcatheter pulmonary valve implantation (TPVI) represents a valid therapeutic option (7). TPVI is minimally invasive compared to surgical pulmonary valve implantation and has been reported as a feasible procedure with successful implantation rates greater than 95% (5, 7). However, it can present challenges due to the anatomical variability of the RVOT and pulmonary artery (PA) (5, 8). Due to the variability in patient anatomy, TPVI requires 3-dimentional (3D) and personalised procedural planning. Patients usually undergo pre-operative computed tomography (CT) scan, and cardiac magnetic resonance (CMR). Recently, innovative rendering systems, such as Mixed Reality (MxR), have been introduced into the market, offering new visualization capabilities, and enhancing the 3D perception of rendered information (9). MxR exploits dedicated headsets overlaying virtual contents directly on the user field of view (e.g., Microsoft Hololens, Magic Leap), with a 3D stereoscopic projection replicating a holographic experience (10). In cardiovascular medicine, MxR technology creates stereoscopic images by combining the three-dimensional virtual model reconstructed from preoperative clinical images (3D echography, CT scan, CMR ± 4D flow), with a real-world surface, therefore enhancing the visualization and navigation of 3D anatomical structures with a patient-specific approach (11) (Figure 1).

**Figure 2:**
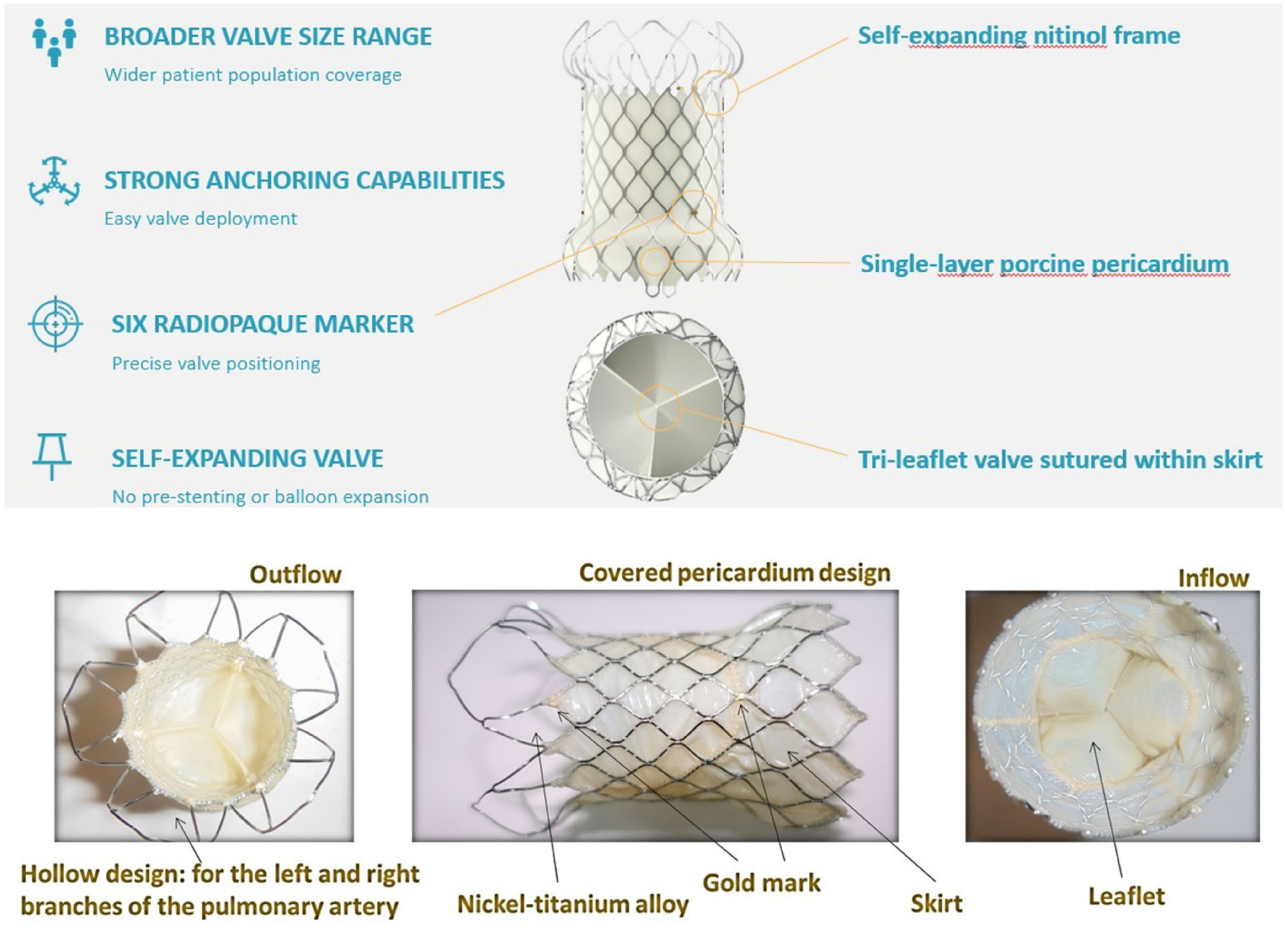
IV generation of flared Venus *P*-valve™.

TPVI is commonly performed in previously placed conduits with bioprosthetic valves of appropriate size (12). More recently, TPVI has been used for the treatment of the native patched RVOT, as devices that accommodate a larger dimension have become available (13). Self-expanding systems provide valve competence despite significant dilatation of the native RVOT (8). For this reason, self-expandable valves have been developed and recently tested internationally and in Europe (14). The Venus *P*-valve™ (Venus Medtech, Hangzhou, China) is a self-expandable bioprosthetic valve that was granted CE-Mark approval under EU MDR (2017/745) for treating patients with pulmonary regurgitation (PR) in the setting of native outflow tracts on April 8, 2022. It is available in two different designs; the straight design has been used for conduits, and the flared one is more appropriate for the treatment of the native RVOT previously repaired with transannular patches (14, 15). The flared Venus *P*-valve consists of a self-expanding nitinol frame and a tri-leaflet valve sutured to a scalloped skirt (Figure 2). The valve leaflets are made of porcine pericardium preserved in low-concentration solutions of buffered glutaraldehyde (15). The frame has proximal and distal flares to anchor the valve in the RVOT and PA bifurcation. The proximal flare is completely covered by pericardial tissue, whereas the distal flare is open to allow access into the PA branches. In newer valve generations, there are six radiopaque markers to identify the valve location during implantation. The diameters of the middle part range from 28 to 36 mm (within 2 mm increments). The device is available in two lengths, 25 and 30 mm. The Venus *P*-valve size must be appropriate to fit the patient's anatomy. It is recommended that the device's middle section diameter is 2–4 mm over size of the main pulmonary artery (MPA). The waist of the MPA is measured by the sizing balloon and the length is equal to or less than the distance from RVOT to PA bifurcation, as measured by fluoroscopy. However, pulmonary arteries have many configurations other than a uniform tubular shape. Therefore, the clinicians should make the final decision based on the anatomy of the patient, with 2–4 mm over sizing in mind for securing the valve implant and preventing peri-annular leak. The transcatheter implantation of the Venus *P*-valve involves the opening of the distal valve flare into one of the PA branches, usually the LPA but also the right pulmonary artery (RPA), to guarantee valve stability during and after the device deployment (15). For this reason, the presence of a previous LPA stent has been reported as a relative contraindication to Venus *P*-valve implantation (14).

## Aim and objectives

This case series aims to describe our experience of Venus *P*-valve implantation in patients with LPA stents and the use holographic models to facilitate procedural planning in these complex clinical cases.

The objective determinants related to the use of patient-specific holographic reconstructions for procedural planning in this patient population could be summarised as follows:

1. Understanding the anatomical morphology of the heart structures
2. Investigating the relationship between LPA, RPA, MPA and valve landing zone
3. Gaining confidence in performing a high-risk procedure by simulating it on the virtual model

## Methods

Monocentric retrospective clinical study approved by the ethics committee.

### Patients

**Graph 1:**
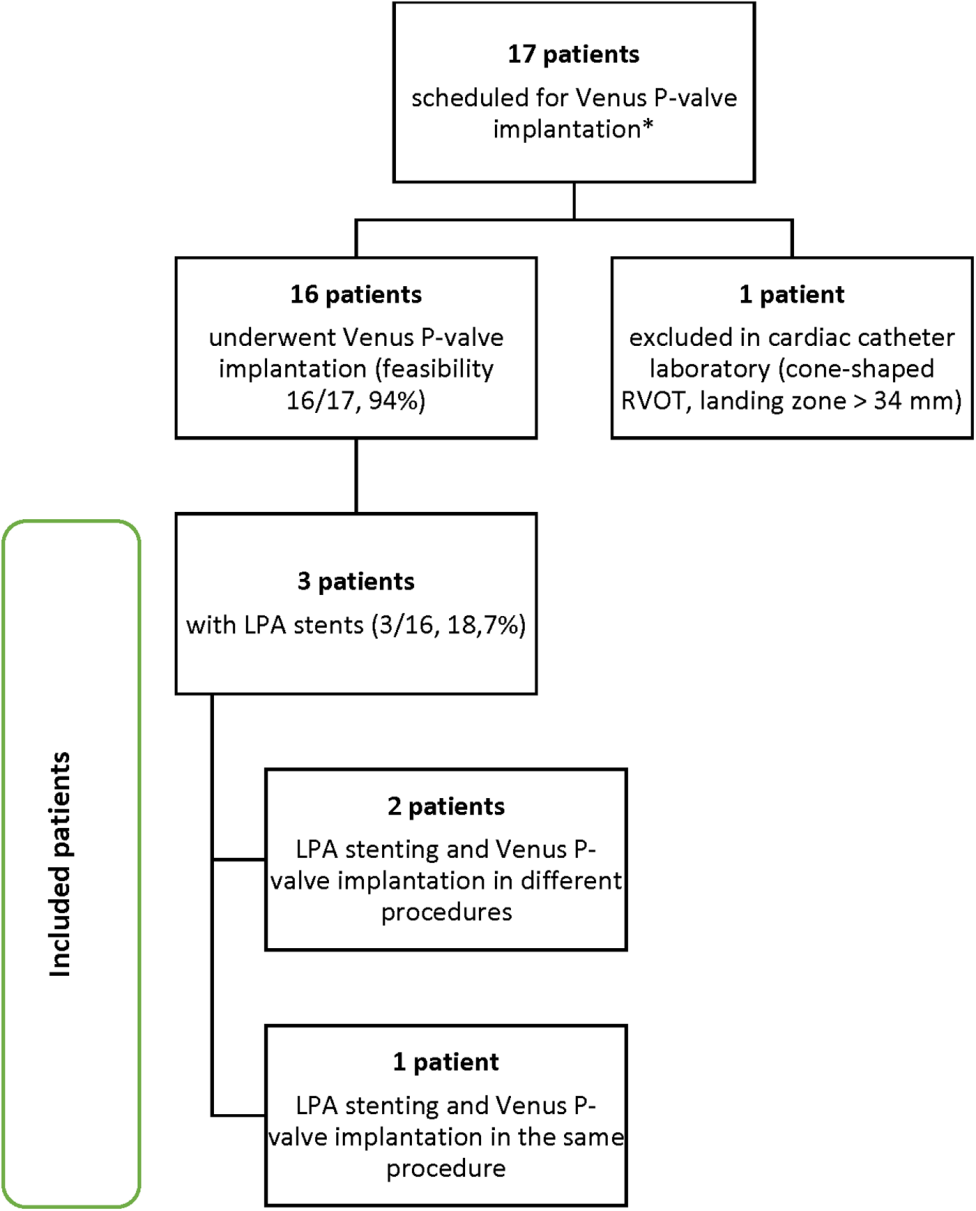
Patient selection process. *All 17 patients were previously discussed with the Venus Medtech specialist team and considered suitable for Venus *P* valve implantation.

From January 2023 to October 2023, a total of 17 patients admitted to our centre were scheduled for Venus *P*-valve implantation, after discussion with the Venus Medtech specialist team. Out of the total 17 patients, 16 patients actually underwent Venus *P*-valve implantation, reaching a procedural feasibility of 94% (*n* = 16/17). One patient, who was initially classified as suitable, was assessed during cardiac catheterisation, and found not to be suitable for Venus *P*-valve implantation, because he had a cone-shaped RVOT, with a landing zone greater than 34 mm. According to the Venus *P*-valve instructions for use, a cone-shaped MPA may cause the valve to fall into the right ventricle. Procedural success in suitable patients was 100% (*n* = 16/16). No mortality or serious post-procedural complications were reported. Three patients (18.7%, *n* = 3/16) underwent Venus *P*-valve implantation with previous LPA stents and were selected for inclusion in this study (Graph 1). Two patients underwent LPA stenting and, after a few months, went back to the cardiac laboratory for Venus *P*-valve implantation; and one patient underwent LPA stenting and Venus *P*-valve implantation in the same procedure. The three patients were selected for inclusion according to the eligibility criteria displayed in Table 1. These criteria are based on the ESC guidelines for the management of adult congenital heart disease (ACHD) (16) and the Venus *P*-valve instructions for use.

**Table 1:** Eligibility criteria.

Inclusion criteria	Exclusion criteria
Age 12–70 years old	Contraindication to anticoagulants
Weight ≥30 kg	Septicaemia
Heart failure symptoms (NYHA ≥ 2)	Recent myocardial infarction (<30 days)
Severe PR (TTE ≥ 3 + or CMR PRRF > 30%)	Intracardiac mass, thrombus, vegetation
If asymptomatic, RVEF < 45%, PRRF > 30%, and RVEDVI > 150 ml/m^2^	Any contraindication of extracorporeal assist
LPA stent prior to Venus *P*-valve implantation	Recent CVA
	Obstruction of central veins
	Bleeding/coagulopathy
	CCR < 20 ml/min
	Pregnancy/breastfeeding
	Known allergies to porcine materials

NYHA, New York Heart Association; PR, pulmonary regurgitation; TTE, trans-thoracic echocardiography; CMR, cardiac magnetic resonance; PRRF, pulmonary regurgitant fraction; RVEF, right ventricle ejection fraction; RVEDVI, right ventricular end-diastolic volume index; LPA, left pulmonary artery; RPA, right pulmonary artery; SPECT, single-photon emission computed tomography; CVA, cerebral vascular accident; CCR; Creatinine Clearance Rate.

### Patient demographics

The patient age range was 20–63 years old. Two patients were females, and one patient was a male. The patients' diagnoses were Tetralogy of Fallot (TOF) or Fallot-type double outlet right ventricle (DORV). The detailed pre-procedural patient characteristics are listed in Table 2. Patient 1 was diagnosed with TOF in neonatal age and underwent palliative treatment with left modified BT shunt when she was 19 months. Then, she was operated at 3 years old, undergoing ventricular septal defect (VSD) closure with dacron patch and RVOT reconstruction with transannular heterologous pericardial patch. Patient 2 had a diagnosis of Fallot-type DORV, we do not have any previous surgical records, but, on the basis of CT scan and CMR data, we supposed that his defect was corrected with transannular patch (TAP). Patient 3 was diagnosed with TOF and underwent correction with TAP at the age of 5 years old. All three patients presented with severe PR and significant LPA stenosis.

**Table 2:** Patient characteristics.

Patients	Age	BMI	Diagnosis	Sex	Palliative treatment	Surgery	LPA stent	NYHA class	CMR RV TDV (ml/m^2^)	CMR RV EF (%)	CMR RV TDV/LV TDV	CMR PRRF (%)	CT PV anulus d (mm)	CT mid MPA d (mm)	CT MPA length (mm)	Sizing balloon d (mm)	Venus *P*-valve size	Fluoroscopy time (min)
1	33	34	TOF + hypoplastic LPA	F	Yes	TAP + LPA plasty	Yes	3	123 (216)	37	2.8	57	23 × 32	34 × 39	45	28	30/25	27
2	20	25	DORV Fallot type	M	No	TAP	Yes	2	85	58	2	38	21 × 30	19 × 29	61	29	30/25	31
3	63	25	TOF	F	No	TAP	No	3	164	38	2.1	48	23 × 29	38 × 40	51	28	32/25	45

NYHA, New York Heart Association; CMR, cardiac magnetic resonance; RV, right ventricle; TVD, tele diastolic volume; EF, ejection fraction; LV, left ventricle; PRRF, pulmonary regurgitant fraction; CT, computed tomography; PV, pulmonary valve; d, diameter; MPA, main pulmonary artery; TOF, tetralogy of Fallot; TAP, transannular patch repair; LPA, left pulmonary artery; VSD, ventricular septal defect; DORV, double-outlet right ventricle.

### Clinical evaluation/procedural planning

**Figure 3:**
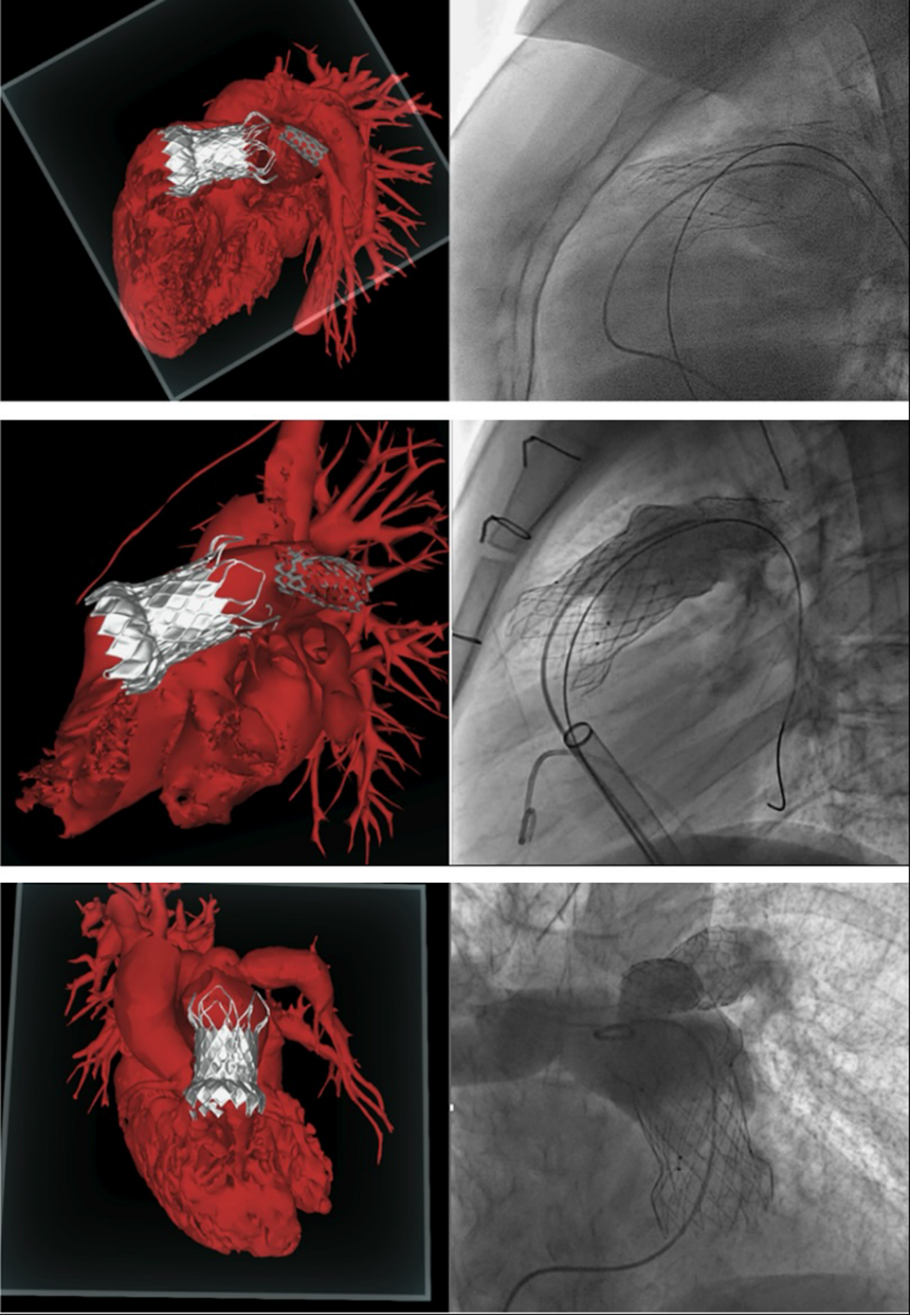
Patients’ holographic models and intra-operative angiograms.

All included patients underwent pre-operative ECG-gated CT scan and CMR. The CT scan data sets of the three patients were analysed by the Venus Medtech team for RVOT dimension and shape, PA branches anatomy, and coronary position in relation to the landing zone. Consequently, a pre-procedural discussion and planning was performed, as it is normally done for all patients scheduled for Venus *P*-valve implantation, in order to confirm procedure feasibility and anticipate intra-procedural risks. The three patients were considered suitable for Venus *P*-valve. However, all of them were categorized as “amber 2”, which is an alert for a high to moderate intra-procedural risk, because an unusual implantation manoeuvre was anticipated due to the presence of the LPA stents. Considering the anticipated difficulty of implantation, CT images were used to create three-dimensional (3D) holographic models (Artiness, Milan, Italy) of the entire heart with a focus on RVOT, PA anatomy, and coronary arteries. The CT segmentation for the holographic reconstruction was done by the bioengineers from Artiness. The holographic models were used for procedural planning by the interventional cardiologists who performed the procedure. During holography-guided procedural planning, dedicated headsets are used to visualize the holographic model of the patient's heart. The interventional cardiologist was then able to interact with the virtual heart, moving it and turning it around, to examine the anatomical structures from different perspectives. In particular, the PA branches and the previous LPA stents were observed in relation to the valve landing zone. The user was also able to use a virtual plan to create sections of the RVOT and take measurements when needed. Furthermore, starting from microCT data of a demo Venus *P*-valve provided by Medtech, the bioengineers of the 3D laboratory at our centre have created a library of holographic Venus *P*-valves in all their dimensions. This is useful because the interventional cardiologist can now simulate the implantation of the Venus *P*-valve of the appropriate size in the patient-specific holographic model, anticipating potential intra-procedural complications (Figure 3).

### Transcatheter procedure

**Graph 2:**
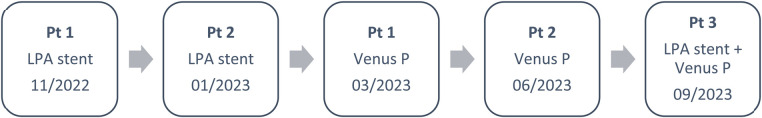
Procedure timeline.

**Figure 4:**
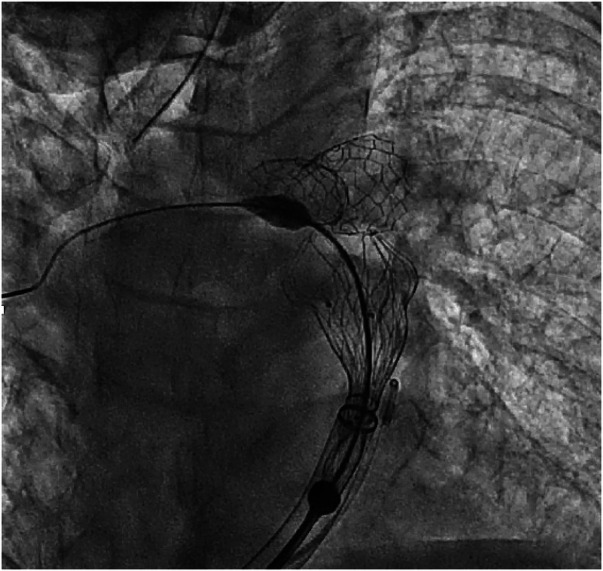
Venus *P*-valve modified implantation technique. The valve was implanted coming from the RPA and flaring in the distal MPA, below the LPA stent.

Included patients were operated in the chronological order displayed in Graph 2. Patients 1 and 2 underwent LPA stenting and Venus *P*-valve implantation in different procedures, whereas for patient 3 this was done in the same procedure. The interventions took place in our cardiac catheter laboratory. All three patients underwent general anaesthesia and were operated via femoral access. 100 IU/kg heparin dose was administered with an additional heparin bolus when needed, maintaining ACT > 250 s throughout. Initially, a diagnostic angiographic catheter was used to take measurements of the RVOT, MPA and PA branches. At this point, the Venus *P*-valve of the appropriate size was selected. Then, an extra stiff guidewire (Lunderquist) was positioned distally into the RPA. A 26 Fr long/sheath (DrySeal GORE) was also advanced distally into to the RPA. The delivery system of the previously selected Venus *P*-valve was subsequently advanced into the long-sheath. The valve was deployed coming from the RPA but starting to open the distal valve flare into the MPA trunk, just below the LPA stent (Figure 4). This implantation technique avoided valve infolding, impingement, and dislocation. After valve deployment, stent patency was confirmed via pulmonary angiography. For patient 3, we placed, as planned, the LPA stent first and then we implanted the Venus *P* valve with the same modified technique used for patients 1 and 2.

## Results

### Clinical outcomes

All procedures were successful and good final haemodynamic and angiographic results were reported. In particular, a good and balanced flow across both PA branches was reported for all three patients. No procedural complications occurred. All three patients were discharged within 72 h from the procedure. The pre-discharge echocardiogram showed no residual transpulmonary gradient at Continuous Wave Doppler (CWD), with no residual pulmonary regurgitation (PR) and/or paravalvular leaks. All patients were discharged with dual antiplatelet therapy (aspirin 100 mg/day and clopidogrel 75 mg/day for 6 months). After 6 months, the pharmacological treatment was changed to aspirin only. One of the patients (patient 3) underwent CT scan due to chest pain the day after the procedure. The CT scan showed no PA dissection, embolism, or effusion secondary to LPA stenting or TPVI. The patient's symptoms were responsive to nonsteroidal anti-inflammatory drugs (NSAIDs) and the pain disappeared after 48 h.

### Follow-up

All patients who undergo TPVI in our centre, undertake several follow-up visits, at 1 month, 6 months, and 12 months. Therefore, all three included patients followed the same clinical pathway. In particular, the 1-month follow-up visit included clinical examination, echocardiography, Holter ECG, and fluoroscopy, in order to exclude stent frame fractures and/or valve or stent dislocation. No patients presented complications at the 1-month follow-up visit. Furthermore, 6 months after the procedure, all patients underwent clinical examination, echocardiogram, Holter ECG, and CT scan. The Holter ECG showed no significant arrhythmias, and positive clinical outcomes were reported by clinical and ultrasound evaluation for all three patients. Moreover, the CT scan at 6 months confirmed the positive procedural outcomes for all three patients. Finally, all patients will undertake a 12-month follow up visit, when they will repeat clinical evaluation, echocardiography, and Holter ECG. Additionally, CMR will also be performed at 12 months.

## Discussion

The aim of this case series was to describe Venus *P*-valve implantation in patients with LPA stents and the use of holographic models to facilitate procedural planning in this patient population. We have included in this case series three patients with previously repaired native RVOT, who were suitable for Venus *P*-valve implantation, and presented severe LPA stenosis or previous LPA stents. The reported frequency of PA abnormalities in patients with TOF is high (18.92%) and the commonest PA abnormality is isolated LPA stenosis (10.4%) (17). In our series of patients who were suitable for Venus *P*-valve implantation, the patients with LPA stenosis or previous LPA stents were 18.7% (*n* = 3/16) of the total. For the first two patients, we decided to perform two separate procedures, the first one for LPA stenting, and the second one for Venus *P*-valve implantation, because we were at the beginning of our experience with Venus *P*-valve in this patient cohort. After gaining confidence with the modified implantation technique, we decided to perform both interventions in the same procedure because we believe that this has the potential to reduce intra-procedural adverse events, such as the exposure to general anaesthesia, radiations, and contrast. For patient 3, we placed the LPA stent first and then we implanted the Venus *P* valve with the same modified technique used for patients 1 and 2. The reason why we decided to proceed in this order, is that we believe that manipulating long sheets inside the valve just after implantation can be compromise valve stability, therefore increasing the risk of valve dislocation and related complications. Venus *P*-valve implantation was successful in all three patients with no procedural complications and good post-procedural clinical outcomes, confirmed at follow-up visits. To our knowledge, there are no other cases described in the literature in which the flared Venus *P*-valve was implanted with pre-stented or after stenting the LPA. Sivakumar et al. (14) reported modifications of the flared Venus *P*-valve implantation technique in patients with narrow LPA. In particular, LPA stenosis in three patients was managed with RPA deployment in two patients and balloon-assisted deployment in one patient (14). These results supported the hypothesis that a narrow LPA should not be considered as an absolute contraindication for Venus *P*-valve. Our case series, not only agrees with this finding, but also demonstrates that even a previous LPA stent can be overcome with careful procedural planning and appropriate implantation technique modifications. In particular, 3D and personalised planning, including the use of holographic models, may have facilitated procedural planning in these complex clinical cases. This is a promising result for a new technology, such as MxR. A recent review on augmented reality (AR) and MxR for healthcare education reported several healthcare educational benefits of both AR and MxR, significantly outperforming traditional learning approaches (18). In particular, AR and MxR were claimed to significantly improve the learning process in all or in the majority of outcome measures, such as the acquisition of anatomy knowledge (18). In a recent study, MxR was evaluated as an effective and engaging tool for undergraduate students learning the anatomy of complex congenital heart disease (CHD) (19). Even if some students complained that the devices were difficult to use, especially at first attempt, they generally agreed that the immersive experience helped them understand the anatomy of the heart structures (19). 3D physical and holographic models have been also used for patient and family education (20). Biglino et al. (21) demonstrated the benefit of patient-specific 3D printed models in the realm of doctor-patient communication, in a group of parents of children with congenital heart disease (CHD). Given the complexity of repaired CHD, a real replica of the area of interest is helpful for the parents to better understand, manipulate, and visualize the anatomical structures, including a specific area that the cardiologist is describing, and what the repair has been or what it will entail (21). Overall, both 3D-printed models and holographic models seem to have the potential to provide significant benefits, including education, training, and improved procedural planning of complex procedures (9, 22). When it comes to procedural planning, the benefits related to the use of holographic models are still to be demonstrated. However, in our experience, it was particularly useful to be able to use personalised holographic heart models, when planning a challenging procedure, such as the Venus *P*-valve implantation in patients with LPA stents. We achieved this “navigating” inside the anatomical structures of the virtual heart, in order to understand the relationship between the origin of the RPA, the origin of the stented/stenotic LPA, and the MPA trunk. In particular, this helped us understanding how to accommodate a self-expandable valve with a large distal flare, such as the Venus *P*-valve, without interfering with the LPA stent. In fact, when accomplishing the transcatheter implantation of the Venus *P*-valve, it is particularly important to consider the length between the pulmonary bifurcation and the pulmonary valve anulus. The possible risks related to the transcatheter implantation of an auto-expandable valve, such as the Venus *P*-valve, in the presence of a LPA stent, include stent dislocation and/or valve impingement in the stent itself. These complications could compromise the success of the procedure. We think that we were able to reduce intra-procedural risks by planning the procedure using patient-specific holographic models. Doing so, have enhanced the perception of the real spatial relationship between the LPA stent and the potential valve landing zone. Finally, our virtual library of Venus *P*-valves was useful to simulate the implantation of the Venus *P*-valve of the desired size in the patient-specific virtual RVOT.

### Strengths and limitations

This study has several strengths and limitations, and the authors believe that it is important to discuss them in relation to the study findings. First of all, we are aware that the study design that we have adopted presents several weaknesses, including the intrinsic risk of patient selection bias, reporting bias, and confounding variables. Furthermore, low generalizability and limited statistical power are inevitable given the small sample size. However, we presented very complex and rare clinical cases, which have never been described in larger studies before. The exploratory insight and the retrospective nature of the study of the study gave us an opportunity to reflect on the real-world clinical practice, offering an insight into how interventions are performed outside of a controlled research setting. This has provided valuable information about the feasibility of interventions in routine clinical care. Moreover, in this case series, we described a pioneer technology, MxR, and its role as procedural planning tool. We believe that the use of holographic models for procedural planning has the potential to reduce procedural time and intra-operative complications. However, to date, the role of pre-procedural planning tools remains limited. Even if the use of MxR can support the interventional cardiologist when studying a complex case, the intra-procedural assessment of tissue characteristics (distensibility of RVOT checked with balloon exploration) and the angiographic RVOT measurements remain central in the decision-making process. Furthermore, the interventional cardiologist's previous experience and confidence may influence patients' outcomes.

## Conclusion

It can be concluded that LPA stenting should not be considered as an absolute contraindication for Venus *P*-valve implantation in native RVOT patients. Careful patient selection and procedural planning are mandatory. Furthermore, the use of holographic models may support procedural panning and technique adaptation in this complex patient population. Additional studies are necessary to further investigate the role of MxR in procedural planning of complex procedures in the field of CHD.

## Data Availability

The datasets presented in this study can be found in online repositories. The raw data can be found here: https://zenodo.org/records/10947794. The data has been uploaded under restricted access and are available upon request to the corresponding author.

## Author's note

The authors are members of the European Reference Network (ERN) for rare, low prevalence, and complex diseases of the heart—ERN GUARD-Heart.
